# Recommendations for good clinical practice for DPD bone scintigraphy for cardiac amyloidosis

**DOI:** 10.1097/MNM.0000000000001796

**Published:** 2024-01-26

**Authors:** Kshama Wechalekar, David Hutt, Ann Marie Quigley, Carol Whelan, Pei San Chan, Lucy Hossen, Ian Armstrong, Parthiban Arumugam, William Moody, Ashutosh D. Wechalekar

**Affiliations:** a Department of Nuclear Medicine, Royal Brompton and Harefield Hospitals, London. Part of Guy’s and St. Thomas’ Foundation Trust,; b National Amyloidosis Centre, University College London,; c Nuclear Medicine, Royal Free Hospital, London,; d Nuclear Medicine Centre, Manchester University NHS Foundation Trust, Manchester and; e Queen Elizabeth Hospital Birmingham, University Hospitals Birmingham NHS Foundation Trust, Birmingham, UK

## Purpose

1. Myocardial uptake of the radiotracer technetium-99m-labelled 3,3-diphosphono-1,2-propanodicarboxylic acid (DPD) or similar bone tracers such as HMDP (Hydroxymethylene diphosphonate) pyrophosphate (PYP) has been validated and increasingly used in the non-biopsy diagnostic pathway of ATTR amyloid cardiomyopathy. DPD scintigraphy is most used in the U.K. It offers a highly sensitive tool for detecting this disease and plays a key role in the non-invasive diagnostic workup of these patients. ATTR cardiac amyloidosis is more prevalent than first realised and, with potential novel therapies in the pipeline, its early detection is important.
2. There are approximately 252 nuclear medicine departments in the UK (https://bnms.org/). Most of the departments perform whole-body bone scans for skeletal metastasis. At least half of the cameras have SPECT capability, and a large proportion of the new cameras have the ability to perform hybrid CT in addition to SPECT. Approximately 30 centres in the UK now perform DPD scintigraphy which is practically not very different from bone scintigraphy that has been practised by nuclear medicine departments for many decades.
3. Cardiac amyloidosis is a relatively rare disease and is not well understood due to complex methods of diagnosis, mainly restricted to specialist centre. Recent advancement in imaging, availability of DPD scans and new drugs to treat cardiac amyloidosis have helped to increase its awareness among cardiologists and nuclear physicians. It remians an underdiagnosed entity.
4. Interpreting DPD scan needs an understanding of the main types of amyloidosis, image patterns, pitfalls, knowledge of further laboratory tests to confirm type of amyloid and the ability to refer appropriate patients for management.

The purpose of these recommendations is to fill the knowledge gaps and empower nuclear physicians, radiologists, and cardiologists for appropriate interpretation of DPD scintigraphy in patients with suspected cardiac amyloidosis and recommend referral to specialist centre, when appropriate.

## Background

Systemic amyloidoses are caused by deposition of misfolded proteins as interstitial fibrillar deposits (amyloid deposits) and direct cellular proteotoxicity from the pre-fibrillary oligomers. Progressive multiorgan dysfunction results from ongoing accumulation of protein fibrils that are remarkably resistant to proteolysis. About 35 different proteins have been reported to cause human amyloidoses. Of these, systemic AL amyloidosis (AL) due to the deposition of misfolded immunoglobulin light chains and wild-type transthyretin amyloidosis (wtATTR), due to misfolded native transthyretin deposition, are the two commonest types. AL was considered the commonest but wtATTR is set to overtake AL as the globally commonest type of amyloidosis. Cardiac involvement remains the most common cause of morbidity and mortality in patients with systemic AL and ATTR amyloidosis. AL has an incidence of 8–10 cases per million person-years, a median age at diagnosis of 63 yrs., and is a rapidly fatal disease, if untreated. Secondary amyloidosis (AA) is becoming rarer in developed countries but still occurs with autoimmune or inflammatory diseases such as multicentric Castleman’s disease, renal cell cancer, autoimmune disorders, and chronic infections due to bronchiectasis or osteomyelitis. Leucocyte chemotactic factor 2 (LECT2) amyloidosis, seen in countries like Mexico, India, and Egypt, is becoming another frequently recognised acquired amyloidosis.

### Clinical manifestations

The heart and kidneys are the two commonest organs affected by amyloidosis. Symptoms due to dysfunction of these organs underpin the clinical presentation – fatigue, breathlessness, and oedema. Wild type ATTR amyloidosis typically presents in an older patient (more frequently male) with symptoms of progressive heart failure. Heart failure with preserved ejection fraction (HFpEF) is typically seen initially in patients with wtATTR amyloidosis but with disease progression, heart failure with reduced ejection fraction will develop. Carpal tunnel involvement (often bilateral) is common and may occur some years before the heart failure symptoms are clinically manifested. Biceps tendon rupture is another typical, but less frequent occurrence, in wtATTR amyloidosis. Many patients have symptoms of spinal canal stenosis but the true prevalence, in an older population with ongoing often unrelated back problems, remains uncertain. The TTR V142I (formerly V122I) mutation is prevalent in the black population (4%) and predisposes to the development of hereditary ATTR cardiac amyloidosis – the clinical syndrome overlaps that of wtATTR amyloidosis although the disease phenotype may be more aggressive. Neuropathic ATTR amyloidosis (the commonest mutation in the UK is transthyretin mutation T80A (formerly T60A) seen in the Irish population; V50M (formerly V30M) is the commonest variant globally) can present with a combination of peripheral and autonomic neuropathy; cardiac involvement is also common, especially in older patients (Gillmore *et al*. Adv Ther. 2022). In AL, soft tissue involvement leading to macroglossia, with a typical stiff enlarged tongue with teeth indentation, is a pathognomonic feature. Peri-orbital purpura (so-called ‘panda’ eyes) in a patient with monoclonal gammopathy puts amyloidosis at the top of the differential diagnoses unless proven otherwise. Involvement of peripheral or autonomic nerves is seen in 20–30% of patients with AL amyloidosis [1]. Unexplained weight loss, diarrhoea, erectile dysfunction, painful/painless peripheral neuropathy may also underlie gastrointestinal, autonomic, and peripheral nerve damage, respectively, in these patients. Whilst some clinical presentations maybe very typical of AL amyloidosis, most often clinical presentations are similar for all types of systemic amyloidosis and often not specific enough to distinguish amyloid fibril type clinically. With the increasing recognition of wtATTR amyloidosis in the elderly, a population group where monoclonal gammopathy of uncertain significance is also common, the potential for two amyloid-forming precursor proteins in a single patient is an increasingly frequent situation in clinical practice, leading to a critical need for accurate amyloid typing in the affected organ. This is often the case in older men with an African descent where gammopathies are more common and 4% of the population carry the V122I TTR variant which also predisposes to amyloidosis.

### Diagnosis

Except for ATTR diagnosed with bone scintigraphy in the absence of a monoclonal dyscrasia (Gillmore *et al*. Circulation 2016), most patients with systemic amyloidosis require a biopsy to confirm amyloid deposition and the protein type in amyloid fibrils. Biopsy of the affected organ has the highest chance of yielding a positive result but may be fraught with risks such as bleeding; hence, abdominal fat or bone marrow biopsies may be a lower-risk alternative. The combination of abdominal fat biopsy and bone marrow biopsy is likely to yield a positive result in 87% of cases with probable AL amyloidosis. Demonstration of amyloid deposition by classic Congo red staining and green birefringence under polarised light remains the gold standard, followed by confirmatory fibril typing. Immunohistochemistry and laser capture with mass spectrometry (LCMS) are both used, the latter is considered to be the gold standard due to high sensitivity and specificity. In LCMS, Congo red–positive areas were dissected from slides, digested, and analysed All amyloid deposits contain Apo E and serum amyloid P protein, which are considered to be amyloid signature proteins. After mass spectrometry, using protein reference databases, amyloid fibril protein can be reliably identified in the vast majority of the cases where adequate tissue samples are available. DNA sequencing of genes related to hereditary variants is also important for confirming proteomic findings with family screening when appropriate.

Direct imaging of amyloid deposits is difficult but, when possible, may give important information about the extent of the disease. Most imaging is an indirect measurement of organ function (echocardiography) or structure (MRI). Imaging is important in assessing prognosis for patients with amyloidosis. Echocardiography with measurement of global longitudinal strain is a widely available tool that can be used in routine clinical practice. Cardiac MRI can give detailed structural information about amyloid heart and provide prognostic information. It also appears to be useful to serially track cardiac amyloid burden by quantification of extracellular volume. However, patients with implanted devices can present imaging challenges and not all centres will administer gadolinium-based contrast agents to patients with impaired renal function because of a theoretical risk of nephrogenic systemic fibrosis. Radionuclide imaging using ^123^I labelled serum amyloid P component scintigraphy can serially monitor changes in the total body amyloid load – and can act as a useful guide to therapy. however, this is not widely available. PET scan using tracers such as Florbetpir or Florbetaben appear to be highly sensitive and specific for cardiac imaging in AL. Confirmatory prospective studies are underway.

### Treatment options

AL and ATTR as the two common types with completely different treatment options. AL is caused by an underlying plasma cell clone and treatment is presently with combination chemotherapy or chemo-immunotherapy with goal of eliminating the clone and reducing the production of the amyloidogenic precursor free light chains. Some patients are candidates for an autologous peripheral blood stem cell transplant. There is absolutely no role for chemotherapy in ATTR amyloidosis. In all types of ATTR amyloidosis (hereditary or wild type), treatment focuses on either stabilising the TTR molecule to reduce the instability or target TTR production in the liver with novel focussed gene silencing therapies. Monoclonal antibody (mAb) treatments directed at accelerating the removal of amyloidosis remains an area of active research. The national amyloidosis centre in UK (NAC) has a pivotal role in clinical trials in AL and ATTR amyloidosis, exploring the respective treatment approaches as well as amyloid imaging and anti-fibril strategies.

### Indications for cardiac imaging for amyloidosis

Appropriate cardiac imaging in amyloidosis is crucial for early and accurate diagnosis of amyloidosis. The first two crucial steps are to undertake imaging to confirm the presence of amyloid deposition in the heart and then the type of amyloid fibrils. Initial investigation with echocardiography (including assessment of diastolic function and global longitudinal strain – a useful marker to differentiate amyloid cardiomyopathy from other types of hypertrophic cardiomyopathy) is appropriate. Echocardiography has an important role in raising the suspicion of cardiac amyloidosis although findings can often be non-specific and so a diagnosis cannot be made without additional confirmatory tests. In patients with unclear pathology on echocardiography or where there is any suspicion of dual pathology, cardiac MRI with gadolinium contrast is indicated. The role of bone scintigraphic tracers is crucial to avoid unnecessary biopsies in the older patients with wtATTR cardiac amyloidosis. It is neither a primary tool to diagnose amyloid cardiomyopathy nor a tool to exclude cardiac amyloidosis on its own.

It is estimated that 95% of all cardiac amyloidosis is ATTR or AL sub-types. The presence of a negative DPD scan effectively excludes ATTR cardiac amyloidosis (with the exception of very rare hATTR variants) but patients with AL cardiac amyloidosis will frequently demonstrate no myocardial DPD tracer uptake. Ideally, all AL cardiac amyloidosis patients should therefore undergo detailed echocardiography or CMR. CMR is also useful in ATTR patients because it provides prognostic as well as diagnostic information. The suggested combination, therefore, is- echocardiography followed by bone scintigraphy, or CMR followed by bone scintigraphy, or both (echocardiography + CMR) followed by bone scintigraphy. All patients undergoing bone scintigraphy for cardiomyopathy *must* have serum and urine immunofixation as well as serum free light chains tested to confirm or rule out presence of a monoclonal protein [2].

**Fig. 1:**
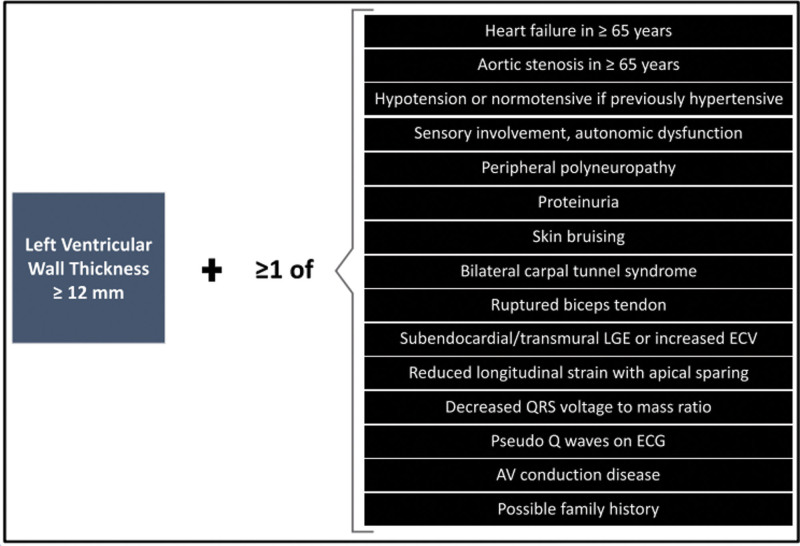
When to suspect a diagnosis of cardiac amyloidosis? AV, atrioventricular; ECG, electrocardiogram; ECV, extracellular volume; LGE, late gadolinium enhancement. Reproduced with permission from the ESC position statement [3].

Almost all patients with cardiac amyloidosis will present with a heart failure syndrome (progressive breathlessness, peripheral oedema, pleural effusions) at which stage most will undergo a transthoracic echocardiogram. This may help raise a suspicion of cardiac amyloidosis (Fig. 1) but bone scintigraphy for cardiac amyloidosis should be considered in the presence of:

1. HFpEF in older patients, particularly in men
2. Heart failure in patients with history of carpal tunnel syndrome or biceps tendon rupture (ATTR)
3. Unexplained heart failure in a patient with monoclonal gammopathy or multiple myeloma (ATTR/AL/both)
4. Heart failure in the presence of autonomic and/or peripheral neuropathy (hATTR/AL) or gastrointestinal symptoms such as alternating constipation/diarrhoea (hATTR/AL)
5. Heart failure with a history of pacemaker, AV conduction disease and/or atrial fibrillation (ATTR)
6. Heart failure with a disproportionately increased NTproBNP and persistent mild elevation of hsTroponin (ATTR/AL)
7. Heart failure with disproportionate left ventricular wall thickening in elderly patients with a history of aortic stenosis (ATTR).

### DPD scintigraphy

This can be performed in any nuclear medicine department with the ability to perform whole-body scans preferably with SPECT or SPECT-CT in addition. The clinicians/cardiologists suspecting cardiac amyloidosis can refer patients for a 99mTc-DPD scan when deemed appropriate for subsequent justification and authorisation by locally entitled IR(ME)R practitioners/authorising operators for the study. There is no special patient preparation, but it is important that patients are provided with information regarding what to expect during their attendance, including contact details for any queries to be raised before the date of appointment. Pregnancy and breastfeeding status checks must be made prior to radiation exposure in accordance with employer’s procedures to ensure compliance with IR(ME)R. If the patient is breastfeeding advice regarding breastfeeding restriction should be issued in accordance with the Teceos DPD product literature (4-hour breastfeeding restriction with expression and discarding of breastmilk during the restriction period) or as advised by the local Medical Physics Expert (MPE).

### Radiopharmaceutical

Various bone-seeking radiotracers have been used for cardiac amyloid evaluation with bone scintigraphy. 99mTc labelled DPD and HMDP are used in Europe and the UK and 99mTc-PYP is used in the USA.

Variation in diagnostic performance among the three tracers has been reported, most likely due to different patient populations and disease status, or imaging protocols. The three tracers are considered interchangeable since there are no definite data to suggest superiority of one tracer over the other. Most importantly, the widely used bone tracer ^99m^Tc -MDP shows a low sensitivity for cardiac ATTR with unknown mechanism and should not be used for this indication.

DPD made by Teceos is not licensed by the marketing authorisation holder, Cis Bio international, for cardiac amyloid imaging nor is it licensed for bone scintigraphy in the UK. There are no available products that are licensed for the cardiac amyloid indication. To introduce an unlicensed product into NHS Trusts, a Drugs and Therapeutics Committee application and approval may be required. It is good practice to have a disclaimer explaining the reason and justification for using the unlicensed product which is signed by the ARSAC (Administration of Radioactive Substances Advisory Committee) practitioner licence holder to confirm their awareness and responsibility for the clinical use of the product, for example, reporting of adverse drug events, holding record of patients administered with the product. The practitioner needs to apply to ARSAC with evidence of training and experience in order to obtain an ARSAC Practitioner licence for using 99mTc DPD for cardiac amyloid imaging. In addition, the ARSAC employer licence for the hospital site where DPD studies are to be performed must be varied (by application to ARSAC) to include 99mTc DPD for cardiac amyloid imaging. Once trained to a satisfactory standard under the practitioner, the operators can use DPD in daily practice as per local protocols.

### Imaging procedure

Good image quality can be achieved with an optimally functioning gamma camera with SPECT and additionally low dose CT functionality (if available) and observing the following guidance:

1. ARSAC diagnostic reference level (DRL): 700MBq. Typical radiopharmaceutical dose range: 630–700 MBq. Lower local DRLs can be established with guidance from the MPE [4].
2. Using LEHR collimators, perform delayed whole-body imaging at 3 h followed by SPECT-CT of the heart. Scanning should start as close to 3 h post-injection (± 10 min) as possible. The SPECT-CT scan should be started soon after completion of the whole-body scan. At least SPECT of the heart should be performed as a standard, if SPECT-CT is not available.
3. The CT scan range should be limited to include coverage of the entire heart. The CT requirements should be discussed with the local CT practitioner and CT MPE to formulate a suitable optimised local protocol in advance.
4. Void bladder prior to the scan and remove all metallic objects as per normal bone scan procedure, including small clips on clothing in preparation for SPECT-CT acquisition.
5. For a typical scanning protocol details, please refer to Appendix 1.

### Special considerations

Some patients may have already been placed on fluid restrictions (e.g. 1.5 L/day) to help manage their symptoms. Although we could ask them to drink some fluids between the injection and scan, it is important to remind the patient to not exceed their daily fluid intake.

1. Patients with amyloidosis can suffer from postural hypotension and therefore care should be taken when getting patients off the bed and is advisable to allow the patient to sit on the bed for a moment, before standing, to prevent falls/collapsing.
2. If the full protocol cannot be followed as the patient cannot comply, other potential imaging options should be discussed with the IR(ME)R practitioner. When considering imaging options, effort should be made to attempt to acquire images of suitable diagnostic quality to answer the clinical question. Consideration should be made as to whether the scan is a baseline or follow-up and whether cardiac uptake is visible on planar imaging. Imaging could be limited to static planar (5 min) over the heart as a minimum in a follow-up patient. However, in a baseline patient, planar whole body is necessary. Imaging of the head is not essential, if the patient is claustrophobic or unable to withstand the duration of the scan for any reason. In patients without cardiac uptake on planar imaging, it may not be possible to fully answer the clinical question without a SPECT-CT.
3. If patient is unable to lie flat, imaging could be performed in standing up or sitting down position, if such ability exists on available cameras.
4. If the whole-body images reveal other abnormal sites of uptake that need more detailed imaging, SPECT-CT to cover specific areas in consultation with the supervising practitioner/authorised operator may be performed for additional information.

### Image interpretation specifically for Tc99m DPD imaging

#### Visual grading: scoring, current guidelines and pitfalls

The original visual scale devised by Perugini *et al*. in 2005, categorised planar and SPECT studies into 4 groups, based on tracer uptake within the heart and bone [5]:

‘Score 0, absent cardiac uptake and normal bone uptake; score 1, mild cardiac uptake, inferior to bone uptake; score 2, moderate cardiac uptake accompanied by attenuated bone uptake; score 3, strong cardiac uptake with mild/absent bone uptake’ (Perugini 2005).

To determine whether a study is truly negative (Perugini grade 0), it is essential that a SPECT (ideally SPECT/CT) study is performed 3 h post-injection. Imaging at 3 h is needed to facilitate blood pool clearance. A SPECT/CT aids the reporter to confidently determine whether uptake is truly myocardial or residual ‘blood pool’ activity within the LV cavity, which may be present, even at 3 h post injection (i.e. false positive).

**Fig. 2:**
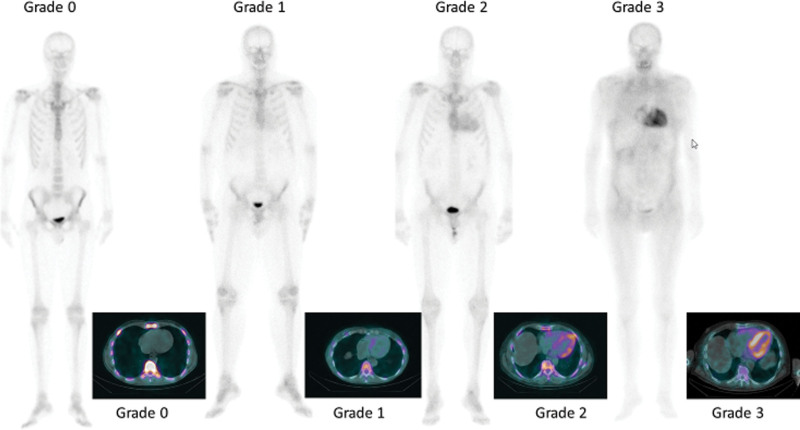
Case examples to demonstrate Perugini grading system and corresponding SPECT-CT images of the heart.

SPECT or SPECT-CT of the heart allows the reporter to identify any low-grade myocardial uptake, that may not be readily appreciated on the planar images as shown in Fig. 2.

1. Low-grade uptake may be demonstrated early in the disease process, and may have implications with regards to patient management, particularly in hereditary TTR variants.
2. Visualisation of cardiac uptake when the myocardial uptake may be obscured by overlying soft tissue uptake on a planar study.

Perugini Grade 1 is uptake within the myocardium that is ‘less than that demonstrated within bone (Perugini 2005). The reference bone is not stated in the original description, but recent guidelines suggest that rib uptake is used as the reference [6]. Bone uptake may be easier to assess on SPECT/CT if there is soft tissue uptake (i.e. tracer uptake within the muscles of the thoracic wall or subcutaneous fat). Vertebral body uptake is however easier to assess than rib uptake, and is more suited to quantification, although active degenerative change should be omitted from ROIs.

Perugini Grade 2 is described myocardial uptake as ‘moderate’. Subsequent guidelines published jointly by ASNC, ASE, EANM, HFSA, ISA, SCMR and SNMMI describe Grade 2 myocardial uptake as equal to that of the ribs [6].

In our expert consensus, a Grade 2 study should demonstrate moderate cardiac uptake ≥ rib. The differentiation between ‘moderate’ and ‘strong’ (i.e. ‘intense’) cardiac uptake is subjective. There may be some attenuation of long bone uptake. Rib uptake is usually preserved (as opposed to a Grade 3 study, in which the rib uptake may become more attenuated, making them difficult to count/ absent altogether on the planar study). As previously mentioned, vertebral body uptake may be used in quantitative methods (Scully et al).

Perugini Grade 3 is described as ‘strong cardiac uptake with mild or absent bone uptake’. The joint guidelines (Dorbala *et al*., 2021) state Grade 3 as ‘uptake greater than rib uptake with mild or absent bone uptake’. Extensive soft tissue uptake may obscure bone uptake, to the extent that the bones (ribs, vertebral bodies, and long bones) become indefinable (i.e. absent) on planar imaging, although they can usually be seen on SPECT/CT, allowing comparison with cardiac uptake.

#### Clinical relevance of visual grading

Contrary to simplistic understanding, up to one-third of AL cardiac amyloid patients may show mild to intense cardiac DPD uptake. Therefore, DPD scan cannot differentiate ATTR from AL amyloidosis by itself. The clinical management of AL amyloid is very different to ATTR amyloid, hence the importance of distinguishing between the two conditions. The tracer may also be taken up within the myocardium in rare non-ATTR amyloid, for example, Apoprotein A1 variant. Cardiac uptake has been reported in myocardial infarction and in the case of Tc99m PYP, associated with hydroxychloroquine toxicity. Focal uptake in the region of the heart on a planar study due to trauma (i.e. rib fractures) can be readily appreciated on SPECT/CT.

#### Additional clinical information

In routine clinical practice, however, differentiating a scan as a Grade 2 or Grade 3, based on the original Perugini description (Perugini 2005) is much less important than differentiating between Grade 1 and Grade 2 scan. Gilmore *et al*. (2016) proposed that Grade 2 or Grade 3 uptake, in the absence of a monoclonal protein in serum or urine, has specificity and positive predictive value of 100% for ATTR amyloidosis (PPV confidence interval of 98–100) and conclude that ‘cardiac ATTR amyloidosis can be reliably diagnosed in the absence of histology, provided that all of the following criteria are met: heart failure with an echocardiogram or CMR that is consistent with or suggestive of amyloidosis, Grade 2 or 3 cardiac uptake on a radionuclide scan with ^99m^Tc-DPD, ^99m^Tc-PYP, or ^99m^Tc-HMDP, and absence of a detectable monoclonal protein despite serum IFE, urine IFE, and sFLC (Freelite) assay. Histological confirmation and typing of amyloid should be sought in all cases of suspected cardiac amyloidosis in which these criteria are not met’. It should be noted however that the Perugini scale was used by Gilmore *et al* [2].

It is not possible to distinguish between hereditary ATTR and wild type on scintigraphy alone. TTR genotyping is required for this differentiation.

**Fig. 3:**
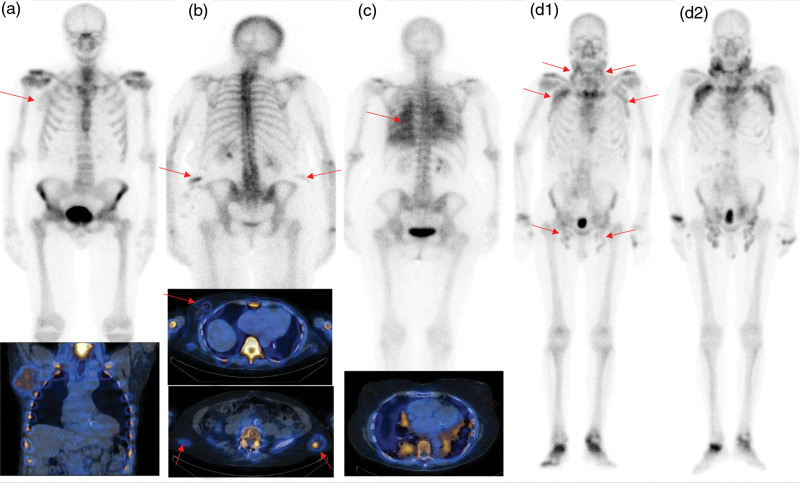
(a–d) Cross-sectional images of ^99m^Tc-DPD (^99m^-Technetium-3, 3,-diphosphono-1,2-propanodicarboxylic acid) uptake in AL patients, by (a) Lymph node, (b) breast and skin, (c) lung parenchyma and (d1) Lymph nodes at baseline and (d2) 25 months later showing progression [7].

It is important to identify extra-cardiac soft tissue sites of uptake on whole body scintigraphy. In a small proportion of patients, moderate to intense uptake of radiolabelled bone tracers by lymph nodes, lung and breast tissue amyloid has been recognised. ^99m^Tc-DPD scintigraphy is a useful imaging modality to detect amyloid in these rare sites and might also be useful for serial imaging (Fig. 3). This technique is particularly useful in patients with IgM-related AL amyloidosis, in which soft tissue amyloidosis accounts for 35% of patients, of whom 20% have lymph node amyloidosis [8].

### Prognostic value of DPD staging

Presently, there is limited utility of DPD scintigraphy in prognosis of amyloidosis. Patients with ATTR amyloidosis without cardiac uptake (i.e. no cardiac ATTR amyloidosis) have better outcomes than patients with cardiac ATTR amyloidosis. 99mTc-DPD scintigraphy is exquisitely sensitive for identification of cardiac ATTR amyloid, but stratification by Perugini grade of positivity at diagnosis has no prognostic significance. Once there is any cardiac uptake on DPD scintigraphy, there is no additional prognostic value of this imaging modality. AL patients with DPD uptake have worse outcomes [9]^.^

### Quantitation of 99mTc-DPD images

Current evidence base suggests a limited role for routine quantification in DPD scintigraphy and this remains an area in need of further research. Quantification from nuclear medicine images is routinely performed to assist with diagnosis and risk stratification. There are two main types of quantification: relative and absolute. Relative quantification commonly comes in the form of ratios, consisting usually of the area of interest and a reference area. This form of quantification is simple and self-normalising hence does not require exact knowledge of the administered activity, scanning protocols or other factors such as patient weight. This technique also does not usually require rigorous corrections to image data such as attenuation or scatter hence can be performed on simple gamma cameras. Absolute quantification aims to calculate the activity concentration or total activity in an organ which can then be normalised to administered activity and patient factors to derive standardise uptake values (SUVs). This requires, as a minimum SPECT-CT acquisition but may also require additional camera functionality to derive SUVs if this is desired.

Relative quantification has been utilised frequently in amyloid imaging on planar images with the most common metric being the heart to contralateral lung (HCL) ratio (1–4). This is measured on the anterior planar image and consists of a region placed over the heart and an equally sized region placed on the contralateral side. Provided regions are equal in size, then the ratio of either the total region counts, or the mean region counts can be used.

The use of SPECT-CT quantification has been demonstrated for ATTR amyloid (5–12) but, to date, this has still not been adopted as routine clinical practice. Challenges include the absence of large multi-centre validation studies and standardisation of the technique, particularly with respect to segmentation of the left ventricle of the heart due to the lack of established software tools.

In the absence of any preferred technique or standardised validated methods quantification remains optional for the diagnosis of cardiac amyloidosis or for use in distinguishing between amyloid types. Further work on quantitative methods and validation should be undertaken in order to address the challenges.

### Referral to UK National Amyloidosis Centre

The UK National Amyloidosis Centre, based at the Royal Free London NHS Foundation Trust and University College London, is a national tertiary referral centre funded by the UK highly specialist commissioning group for diagnosis and management advice for all types of amyloidosis. Any patient with proven or high suspicion of amyloidosis is suitable for referral to the UK-NAC. All patients attending the NAC for assessment will undergo a combination of tests to confirm the diagnosis and assess the extent of amyloid deposits. Baseline investigations for all patients include blood and urine panels for organ function, biomarkers and monoclonal protein assessment, ^123^I-labelled SAP scintigraphy (for amyloid deposition in visceral organs), echocardiography, and, in selected patients, cardiac MRI and genetic sequencing for hereditary amyloidosis. Older patients with suspicion of cardiac amyloidosis also undergo ^99mTc^DPD scintigraphy. Abdominal fat biopsies are undertaken during the visit and other tissue biopsies are requested from the local centres for amyloid fibril typing. The following as suggestions for patient referral:

1. All patients with suspected or confirmed AL amyloidosis, AA amyloidosis, ALECT2 amyloidosis, presumed localised amyloidosis and familial amyloidosis (hATTR, AFib, AApoA1, AApoA2, B2-M, AGelsolin, ALysozyme) should ideally be referred to the NAC for full assessment. Once the assessments complete, they can be started on chemotherapy, if appropraite, and can be monitored with local referring team. There are a number of ongoing trials of chemotherapy and anti-fibril antibodies for which selected patients may be suitable.
2. There is no routine necessity to refer older patients clearly fulfilling the non-biopsy diagnostic criteria for wild-type ATTR amyloidosis. Clinical trials are available for patients with ATTR amyloidosis, but treatment is not yet routinely commissioned in 2023 by the NHS for wtATTT amyloidosis. Treatments are available for neuropathic hereditary ATTR amyloidosis.
3. Any patient with suspected wtATTR or other ATTR amyloidosis with a monoclonal gammopathy (25% of wtATTR patients will have monoclonal gammopathy) could also be referred to the NAC. In any patient with clinical uncertainty about appropriateness of referral or if the patient is too unwell to travel, direct discussion with the NAC team is recommended. More information can be found at https://www.ucl.ac.uk/amyloidosis/national-amyloidosis-centre.

### Sample report

**Table:** 

Demographics	Name, DOB, hospital number etc.
Indication	Increased LV wall thickness and restrictive pattern of filling on echo? Cardiac amyloidosis.
Technique/method	Isotope used, activity injected, time interval post-injection, acquisition (WB, SPECT/CT of heart/FOV/parameters).
Findings	Adequacy/quality of image. Cardiac uptake: Y/N. Use SPECT/CT to determine if uptake is truly myocardial or blood pool. (Thresholding may be required to assess for low-grade i.e., grade 1 uptake on SPECT/CT). Distribution of cardiac tracer uptake: diffuse/focal. Soft tissue uptake/ bone attenuation (often easier to appreciate on WB imaging). Perugini grade: 0,1,2,3 Quantification if validated in your centre. Incidental findings on planar and SPECT/CT
Interpretation	Negative study: No increased tracer uptake within the myocardium. Grade 0. It is important to stress that a negative scan does not exclude the presence of cardiac amyloid. Positive study: in keeping with the presence of cardiac amyloid (Grades 1, 2 and 3). The reporter should make it clear that it is NOT possible to determine whether uptake is due to ATTR or AL amyloid by scintigraphy alone. Recommend serum-free light chain assay + Serum immunofixation + urine immunofixation (to exclude monoclonal gammopathy) Patients who do not satisfy non-biopsy diagnostic criteria – Consider referral to specialist centre, for example, National Amyloid Centre, Royal Free Campus, London for further typing of amyloid and treatment recommendations.

A Grade 2 or 3 scan, in the absence of a serum or urine monoclonal protein or abnormal serum free light chain ratio (i.e. in those patients who do not have a monoclonal gammopathy), is diagnostic of ATTR amyloid cardiomyopathy. If a monoclonal gammopathy is present by any of these tests, histological typing of amyloid is required to establish a definitive diagnosis often via endomyocardial biopsy and referral to a haematologist/cardiac amyloidosis specialist is recommended (Gillmore *et al*., 2016).

Please see case examples in Figs. 4 and 5 to demonstrate importance of performing SPECT-CT below for identifying myocardial uptake.

### Case example 1

**Fig. 4:**
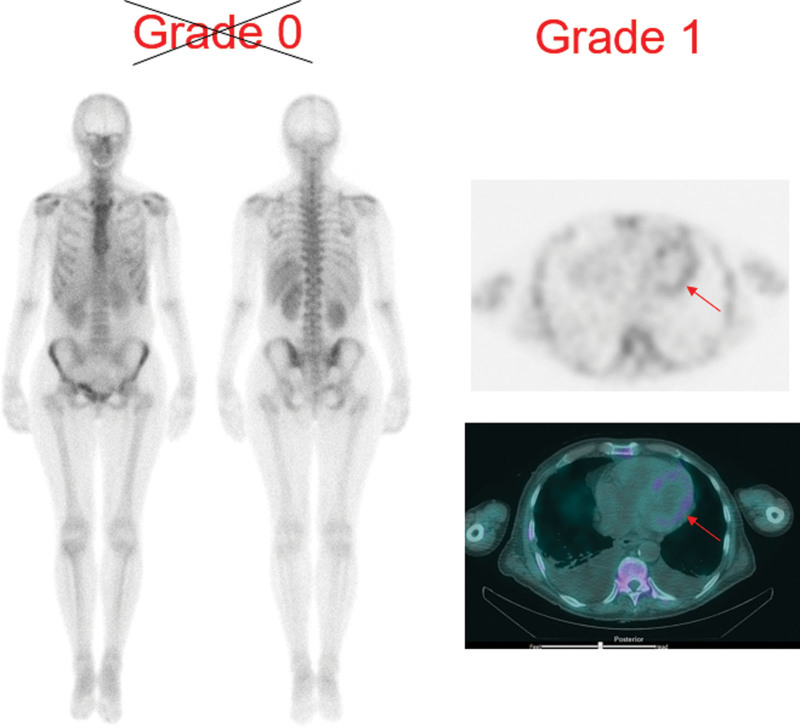
The planar whole body ^99m^Tc-DPD images show diffuse soft tissue uptake in the chest overlapping with uptake in the upper abdominal organs (liver and spleen). No discernible tracer uptake is shown in the region of heart. This would suggest Grade 0 Perugini uptake. However, SPECT-CT images show low grade uptake in the myocardium.

### Case example 2

**Fig. 5:**
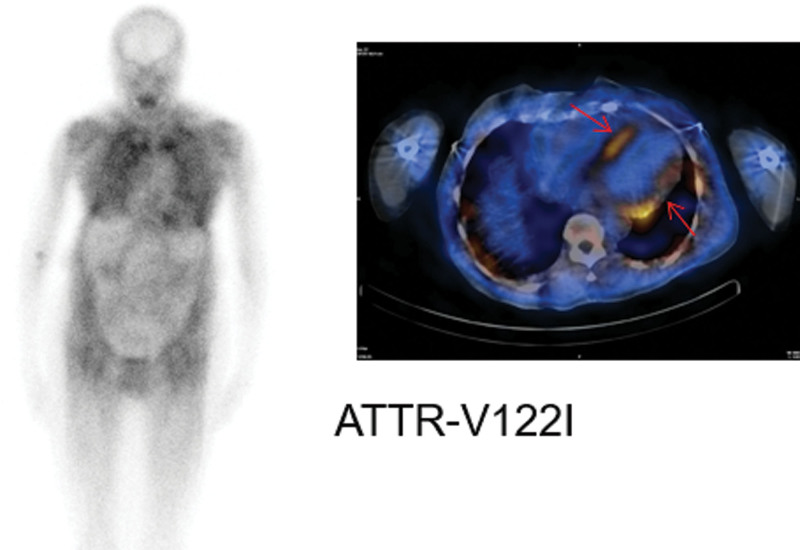
The planar whole body ^99m^Tc-DPD scan images show increased soft tissue uptake in the lungs, muscles of shoulder, pelvic girdle and in the muscles of abdominal wall with poor definition of bones. The cardiac uptake may be present but without the help of SPECT-CT, it would not be possible to be confident about uptake and distribution of cardiac uptake. This patient was diagnosed with ATTR cardiac amyloidosis after biochemical investigations.

## Key messages

1. Cardiac uptake on DPD = Cardiac amyloid (99% of patients with ATTR cardiac amyloid will have cardiac uptake, however, so will 51% of patients with AL cardiac amyloid)
2. No cardiac uptake on DPD ≠ No cardiac amyloid (49% of patients with AL cardiac amyloid will have no cardiac uptake)
3. Recommend serum-free light chain assay + Serum immunofixation+ urine immunofixation (to exclude monoclonal gammopathy) in all patients suspected of cardiac amyloidosis.
4. Referral to specialist centre, for example, National Amyloid Centre, Royal Free Campus, London for further typing of amyloid and treatment recommendations.

## Acknowledgements

Commissioned by British Nuclear Medicine Society, Endorsed by British Cardiovascular Society and British Society for Heart Failure.

### Conflicts of interest

There are no conflicts of interest.

## Appendix 1

Typical Scanning Protocol Details Using the ARSAC DRL: To be adapted for local variations in equipment and DRLs with MPE guidance.

**Table:**

Protocol	Whole-body 3 h
Whole body key parameters	
Detector 1	Ant
Detector 2	Post
Detector head configuration	H mode (opposing)
Body contour	On
Scan Range	Include the whole body- head to toes
Scan mode	Continuous
Exposure time per pixel (s)	169
Palette velocity (cm/min)	14.2
Zoom	1
Pixel size (mm)	2.21
Whole body corrections	
Energy session	Tc-99m
EM	140.5 ± 10%
Collimator	LEHR

SPECT -CT to be performed to cover heart. CT Parameters to be agreed with the local MPE for CT

**Table:**

Protocol	SPECT-CT
Tomo key parameters	
Scan range	Limited to cover just the heart
Patient location	Feet first supine
Gantry mode	H
Body contour	On
Start position (degrees)	0
Zoom	1
Matrix	128 × 128
Scan mode	Step and shoot
Time per view (seconds)	12
Tomo corrections	
Energy session	Tc-99m SC (140.5 and 120)
EM	140.5 ± 10%
SC	120 ± 5%
Collimator	LEHR
COR correction	On
Tomo location parameters	
Table height (cm)	95
Total angular range (degrees)	360
View angle (degrees)	3
Direction	CCW
Detector 1	On
Detector 2	On
Tomo CT/AC parameters	
Series 1	
Scan type	Scout
Voltage (kV)	120
Current (mA)	10
Scout plane (degrees)	180
Series 2	
Scan type	Helical
Voltage (kV)	120
Current (mA)	Smart mA
Min current (mA)	20
Max current (mA)	150
Noise index	35
Slice thickness	3.75
SFOV	Large
CTDI (milli Grey)	5.4

After checking the quality of planar and SPECT-CT scan, the patient can be sent home. Imaging parameters for solid-state cameras may vary. If using dedicated cardiac scanners, please check for imaging and processing protocols with the vendors in advance.
